# Risk factors analysis and nomograph model construction of unplanned readmission for ischemic stroke within 31 days in Wenzhou

**DOI:** 10.3389/fneur.2025.1499564

**Published:** 2025-03-27

**Authors:** Yingying Ma, Zhili Jin, Xianqiong Yi, Xinxin Ye

**Affiliations:** Wenzhou TCM Hospital of Zhejiang Chinese Medical University, Wenzhou, China

**Keywords:** unplanned readmission within 31 days, ischemic stroke, risk model, nomograph, pediatrics

## Abstract

**Objective:**

To investigate independent risk factors for unplanned readmission of ischemic stroke patients within 31 days in Wenzhou, and establish a nomogram model for risk prediction.

**Methods:**

A total of 3,035 patients with ischemic stroke were randomly grouped (in an 8:2 ratio) into 2,428 training set and 607validation set. Independent sample *t*-test, Pearson chi-square test, Fisher’s exact and multivariate logistic regression analysis were used to determine the factors associated with 31-day unplanned readmission in ischemic stroke, and the nomogram was established and validated.

**Results:**

Six hundred and sixty nine patients (22.04%) had unplanned readmission. Atrial fibrillation, smoking, education of junior high school and below, length of stay >16 days, Hcy, and UA were independent risk factors for 31-day unplanned readmission in patients with ischemic stroke. The training set [AUC = 0.883 (95% CI = 0.867–0.899)] and validation set [AUC = 0.817 (95% CI = 0.777–0.858)], and the calibration curve closely resembled the ideal curve, demonstrating good agreement between the predicted and actual values, it shows that the prediction model has a good degree of differentiation and calibration. At the same time, the decision curve shows that the model has a high clinical net benefit rate.

**Conclusion:**

The nomograph model established in this study to predict the risk of unplanned readmission of ischemic stroke patients within 31 days has good prediction ability.

## 1 Introduction

Stroke remains a public health challenge, placing a significant financial burden on healthcare systems among survivors who are disabled as a result of stroke. In Western countries, stroke is the third leading cause of death after coronary heart disease and cancer (1). Ischemic stroke (IS) is the most common type of stroke, and accounts for 80% of the total incidence of stroke. Relative to many other diseases, stroke has a high readmission rate (2, 3). A cohort study in the United States showed that the short-term unplanned readmission rate of cerebral infarction patients was 12.4% (4). Lee et al. (5) showed that 9.2% of patients admitted to hospital for stroke in South Korea were readmitted and 7.6% were unplanned readmitted. A multi-center study in China reported that 28.8% of patients with IS were unplanned readmitted within 31 days of discharge (6).

Unplanned early readmission is an important indicator of hospital medical quality and nursing quality. Stroke incidence varies widely in the north and south regions, in China (7). According to the China Stroke Screening Survey (CNSSS), the incidence of IS in Zhejiang regions has been increasing year by year, and the incidence of IS is higher in males than females, and higher in rural areas than in urban areas (8). However, there is no study on unplanned readmission of IS patients in Zhejiang regions.

Therefore, the purpose of the study was two major aims. First, we investigated the risk factors of 31-day unplanned readmission in ischemic stroke patients, and unplanned readmission rate. Second, to establish a prognostic model for IS patients with unplanned readmission. In recent years, the nomogram as a forecasting method has played an important role in the development of personalized medicine. This study was based on a dataset of clinical studies to establish a nomogram of the risk of unplanned readmission in patients with IS.

## 2 Methods

### 2.1 Data and study population

This retrospective study was conducted at the Wenzhou TCM Hospital of Zhejiang Chinese Medical University between 2020 and 2022. Patients with ischemic stroke were identified using the hospital’s electronic medical record system, using the International Classification of Diseases Code (10th edition) (ICD-10) of the primary diagnostic record. After excluding subjects with missing data, a total of 3,035 patients inclusion in this study, of whom 669 were unplanned readmissions within 31 days of discharge (the unplanned readmission rate was 22.04%).

### 2.2 Interpretation of relevant indicators, inclusion, and exclusion criteria

Unplanned readmitted: refers to the readmitted patients who are unpredictable at the time of discharge, excluding patients: planned readmitted and readmitted with new diseases different from the previous admission.

Inclusion criteria: ① The previous admission was diagnosed IS (ICD-10: I63); ② Age ≥18 years old; ③ The time between last discharge and readmission was within 31 days; ④ readmitted to hospital due to the same or related disease.

Exclusion criteria: ① Death or transfer to hospital after the previous hospitalization; ② Patients was planned to be admitted due to staging surgery, reexamination, and etc.

The selection method for 31-day unplanned readmission cases was first generated through the unplanned readmission report module in the electronic medical record system. Clinicians then examined the cases to determine whether they met the inclusion and exclusion criteria.

### 2.3 Data collection

Clinical data of the patients were collected, including gender, age, BMI (≤24 kg/m^2^, >24 kg/m^2^), marital status (married, divorced, other), residential environment (city, countryside), smoking (no, yes), drinking (no, yes), education (high school and above, junior high school and below), hypertension (no, yes), diabetes (no, yes), hypertension (no, yes), atrial fibrillation (no, yes), operation (no, yes), length of stay (≤16 days, >16 days).

Laboratory examination data including homocysteine (Hcy), glycosylated hemoglobin (HbA1), fasting blood glucose (FBG), uric acid (UA), serumcreatinine (Scr), blood urea nitrogen (BUN), total cholesterol (TC), triglycerides (TG), low-density lipoprotein cholesterol (LDL-C), high-density lipoprotein cholesterol (HDL-C).

This study was approved by the Medical Ethics Committee of the Wenzhou Hospital of Traditional Chinese Medicine and was conducted in accordance with the Declaration of Helsinki.

### 2.4 Statistical analyses

RStudio 4.1.1 and SPSS 25.0 software was used to the part of analyses in study. Continuous variables (normal distribution and uniform variance) were described by $\bar{x\;}\pm s$ , independent sample t-test was used to comparisons between the groups; count variables were described by frequency (percentage), and were compared using the Pearson chi-square test and Fisher’s exact probability test. The variables that were significant in the univariate analysis were included in the multivariate logistic regression analysis to construct the prediction model. The nomogram prediction model was constructed by using RStudio software rms package, and the ability of the model to the predict 31-day unplanned readmission was evaluated by receiver operator characteristic (ROC). The bootstrap method was used to repeat the sampling 1,000 times for internal validation, and the prediction model was evaluated by calibration curves. Decision curves evaluate the clinical value of the prediction model. A *p* < 0.05 indicated that the difference was statistically significant.

## 3 Results

The 3,035 patients were randomly allocated into training (2,428 patients) and validation set (607 patients) in an 8:2 ratio. There was no significant difference in the baseline data of age, gender, marital status education, smoking, drinking, hyperlipidemia, diabetes, hypertension, atrial fibrillation, operation, length of hospital stay between the training set and the verification set (*p* > 0.05) as shown in Table 1.

**Table 1:** Comparison of clinical information of the two group in training set.

Index	Category	Training set (*n* = 2,428)	Validation set (*n* = 607)	*χ* ^2^	*p*
Gender [*n* (%)]	Male	1,577 (64.95)	390 (64.25)	0.104	0.747
	Female	851 (35.05)	217 (35.75)		
Age	≤60	509 (20.96)	119 (19.6)	0.547	0.460
	>60	1,919 (79.04)	488 (80.4)		
Marital status education [*n* (%)]	Married	2,378 (97.94)	588 (96.87)	—	0.146^a^
	Divorced	10 (0.41)	6 (0.99)		
	Other	40 (1.65)	13 (2.14)		
Smoking [*n* (%)]	No	2,296 (94.56)	572 (94.23)	0.101	0.750
	Yes	132 (5.44)	35 (5.77)		
Drinking [*n* (%)]	No	2,353 (96.91)	590 (97.2)	0.137	0.711
	Yes	75 (3.09)	17 (2.8)		
Hyperlipidemia [*n* (%)]	No	1,730 (71.25)	448 (73.81)	1.563	0.211
	Yes	698 (28.75)	159 (26.19)		
Diabetes [*n* (%)]	No	1,497 (61.66)	354 (58.32)	2.271	0.132
	Yes	931 (38.34)	253 (41.68)		
Hypertension [*n* (%)]	No	1,274 (52.47)	305 (50.25)	0.962	0.327
	Yes	1,154 (47.53)	302 (49.75)		
Atrial fibrillation	No	2,248 (92.59)	553 (91.1)	1.500	0.221
	Yes	180 (7.41)	54 (8.9)		
Operation	No	2,334 (96.13)	584 (96.21)	0.009	0.925
	Yes	94 (3.87)	23 (3.79)		
Length of hospital stay	≤16 days	1,278 (52.64)	339 (55.85)	2.013	0.156
	>16 days	1,150 (47.36)	268 (44.15)		

^aindicates the exact probability of using Fisher.^

### 3.1 Comparison of clinical data and laboratory examination between the two groups in the training set

Within the training set, patients were further categorized into a 31-day unplanned readmission group (535 cases) and a non-31-day unplanned readmission group (1,893 cases). There was no statistically significant difference between the non-31-day unplanned readmission group and the 31-day unplanned readmission group in terms of gender, BMI, age, marital status, residential, drinking, hyperlipidemia, diabetes, HbA1c, FBG, Scr, BUN, TC, TG, LDL-C, and HDL-C (*p* > 0.05). In the 31-day unplanned readmission group, patients with smoking, junior high school education or below, hypertension, atrial fibrillation, surgery, and hospital stay >16 days accounted for a higher proportion of patients, and the levels of UA and Hcy were higher than those in the non-31-day unplanned readmission group, with statistical significance (*p* < 0.05) as shown in Tables 2, 3.

**Table 2:** Comparison of clinical information of the two group in training set.

Index	Category	Non-31-day unplanned readmission group (*n* = 1,893)	31-day unplanned readmission group (*n* = 535)	*χ* ^2^	*p*
Gender [*n* (%)]	Male	1,223 (64.61)	354 (66.17)	0.447	0.504
	Female	670 (35.39)	181 (33.83)		
BMI (kg/m^2^) [*n* (%)]	≤24	1,365 (72.11)	364 (68.04)	3.371	0.066
	>24	528 (27.89)	171 (31.96)		
Age	≤60	408 (21.55)	101 (18.88)	1.801	0.180
	>60	1,485 (78.45)	434 (81.12)		
Marital status education [*n* (%)]	Married	1,850 (97.73)	528 (98.69)	—	0.213^a^
	Divorced	10 (0.53)	0 (0.00)		
	Other	33 (1.74)	7 (1.31)		
Residential environment [*n* (%)]	City	1,192 (62.97)	320 (59.81)	1.768	0.184
	Country side	701 (37.03)	215 (40.19)		
Smoking [*n* (%)]	No	1,815 (95.88)	481 (89.91)	28.946	<0.001
	Yes	78 (4.12)	54 (10.09)		
Drinking [*n* (%)]	No	1,840 (97.2)	513 (95.89)	2.400	0.121
	Yes	53 (2.80)	22 (4.11)		
Education [*n* (%)]	High school and above	1,532 (80.93)	382 (68.83)	36.848	<0.001
	Junior high school and below	361 (19.07)	173 (31.17)		
Hyperlipidemia [*n* (%)]	No	1,355 (71.58)	375 (70.09)	0.450	0.502
	Yes	538 (28.42)	160 (29.91)		
Diabetes [*n* (%)]	No	1,166 (61.6)	331 (61.87)	0.013	0.908
	Yes	727 (38.40)	204 (38.13)		
Hypertension [*n* (%)]	No	966 (51.03)	308 (57.57)	7.154	0.007
	Yes	927 (48.97)	227 (42.43)		
Atrial fibrillation	No	1,776 (93.82)	472 (88.22)	19.023	<0.001
	Yes	117 (6.18)	63 (11.78)		
Operation	No	1,829 (96.62)	505 (94.39)	5.557	0.018
	Yes	64 (3.38)	30 (5.61)		
Length of hospital stay	≤16 days	1,191 (62.92)	87 (16.26)	364.173	<0.001
	>16 days	702 (37.08)	448 (83.74)		

a Fisher exact probability test was used for comparison.

**Table 3:** Comparison of relevant laboratory examination of the two group in training set.

Index	Non-31-day unplanned readmission group (*n* = 1,893)	31-day unplanned readmission group (*n* = 535)	*t*	*p*
Hcy (μmol/L)	15.28 ± 3.66	18.62 ± 4.89	−17.178	<0.001
HbA1c (%)	5.90 ± 1.90	6.00 ± 2.02	−0.222	0.825
FBG (mmol/L)	5.89 ± 1.75	5.92 ± 1.70	−0.367	0.714
UA (μmol/L)	331.04 ± 43.62	376.67 ± 55.44	−20.050	<0.001
Scr (μmol/L)	89.39 ± 12.86	89.87 ± 14.33	−0.738	0.460
BUN (mmol/L)	5.68 ± 1.07	5.71 ± 1.04	−0.475	0.635
TC (mmol/L)	4.90 ± 1.53	4.95 ± 1.39	−0.723	0.470
TG (mmol/L)	1.62 ± 0.39	1.66 ± 0.66	−1.934	0.053
LDL-C (mmol/L)	2.86 ± 0.86	2.91 ± 0.88	−1.072	0.284
HDL-C (mmol/L)	1.21 ± 0.15	1.19 ± 0.16	1.901	0.057

Hcy, homocysteine; HbA1, glycosylated hemoglobin; FBG, fasting blood glucose; UA, uric acid; Scr, serumcreatinine; BUN, blood urea nitrogen; TC, total cholesterol; TG, triglycerides; LDL-C, low-density lipoprotein cholesterol; HDL-C, high-density lipoprotein cholesterol.

### 3.2 Multivariate logistic regression analysis of stroke patients with unplanned readmissions within 31 days

In the multivariate analysis, significant factors found were atrial fibrillation (OR = 1.811; 95% CI: 1.178–2.784), smoking (OR = 2.571; 95% CI: 1.896–4.924), education of junior high school and below (OR = 2.021; 95% CI: 1.533–2.664), length of stay >16 days (OR = 8.793; 95% CI: 6.603–11.709), Hcy (OR = 1.238; 95% CI: 1.199–1.279), and UA (OR = 1.020; 95% CI: 1.018–1.023), as shown in Table 4.

**Table 4:** Multiple logistic regression analysis of 31-day unplanned readmission in ischemic stroke patients.

Index	Category	*β*	SE	Wald	*p*	OR	95% CI
Hypertension	No					1	
	Yes	0.292	0.213	1.882	0.170	1.339	0.882–2.033
Atrial fibrillation	No					1	
	Yes	0.594	0.219	7.321	0.007	1.811	1.178–2.784
Operation	No					1	
	Yes	0.355	0.292	1.484	0.223	1.427	0.805–2.527
Smoking	No					1	
	Yes	1.117	0.244	21.033	<0.001	2.571	1.896–4.924
Education	High school and above					1	
	Junior high school and below	0.704	0.141	24.912	<0.001	2.021	1.533–2.664
Length of stay	≤16 days					1	
	>16 days	2.174	0.146	221.369	<0.001	8.793	6.603–11.709
Hcy		0.214	0.016	168.635	<0.001	1.238	1.199–1.279
UA		0.020	0.001	208.181	<0.001	1.020	1.018–1.023
Constant		−18.027	0.877	422.248	<0.001		

Hcy, homocysteine; UA, uric acid.

### 3.3 Construction of a nomograph model

**Figure 1:**
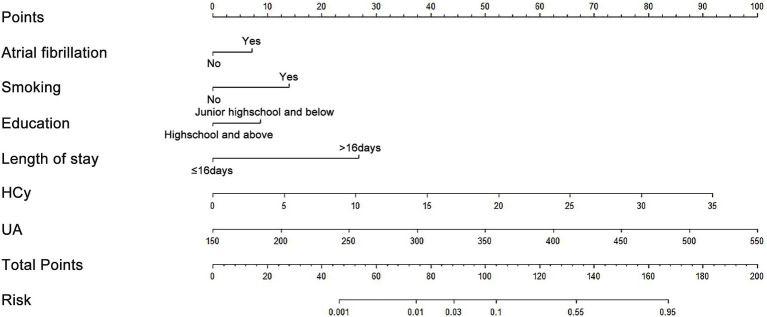
Nomogram model for predicting the risk of 31-day unplanned readmission in ischemic stroke patients.

Based on logistic regression results, a nomogram was constructed to determine the risk of 31-day unplanned readmission of IS patients after discharge. In the nomogram, the total score corresponding to each independent influencing factor is added to calculate the total score, and the predicted probability of 31-day unplanned readmission corresponding to the total score is obtained (Figure 1).

### 3.4 Test the predictive efficiency of the nomogram model

**Figure 2:**
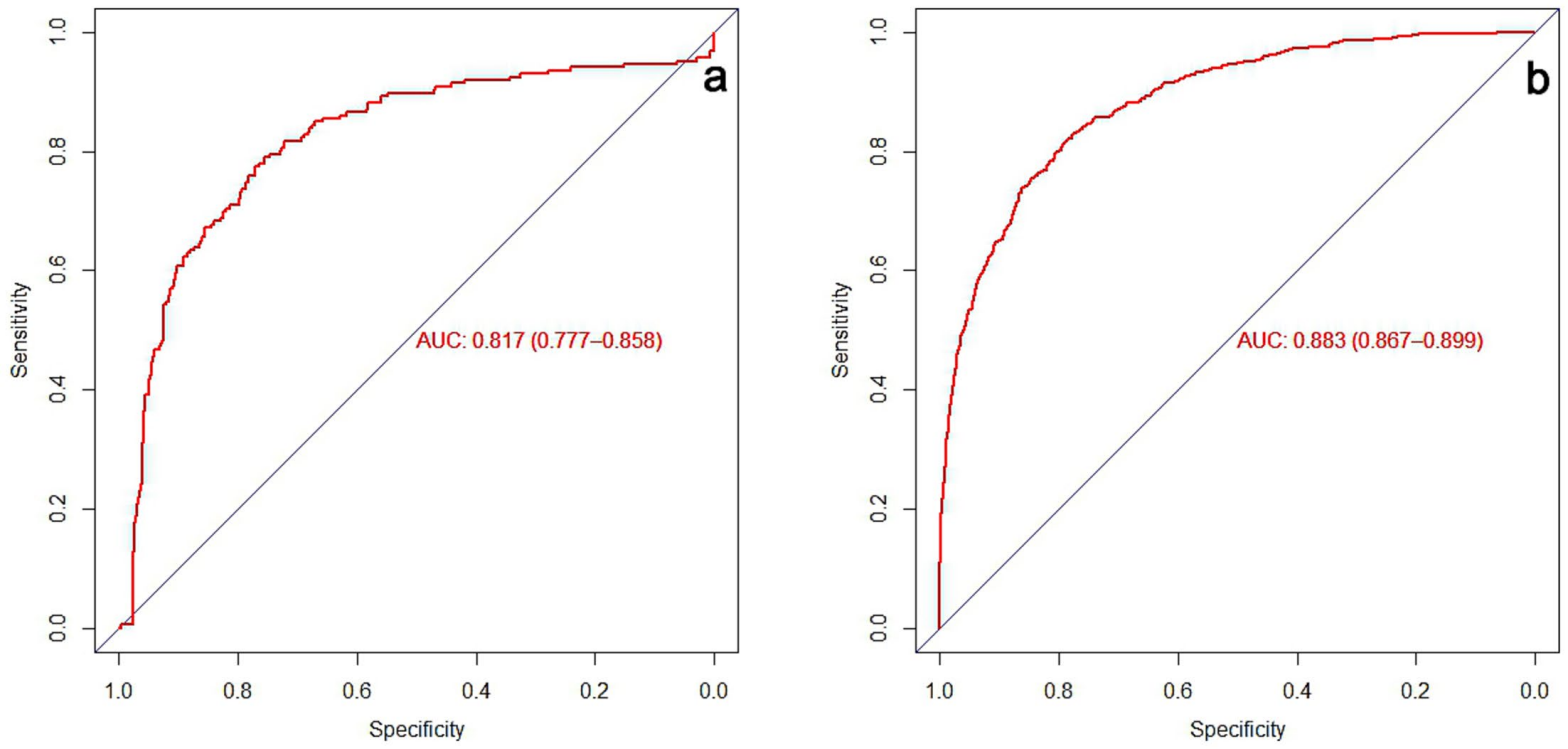
Prediction of 31-day unplanned readmission ROC curve for ischemic stroke patients. **(a)** Training set ROC curve. **(b)** Verification set ROC curve.

**Figure 3:**
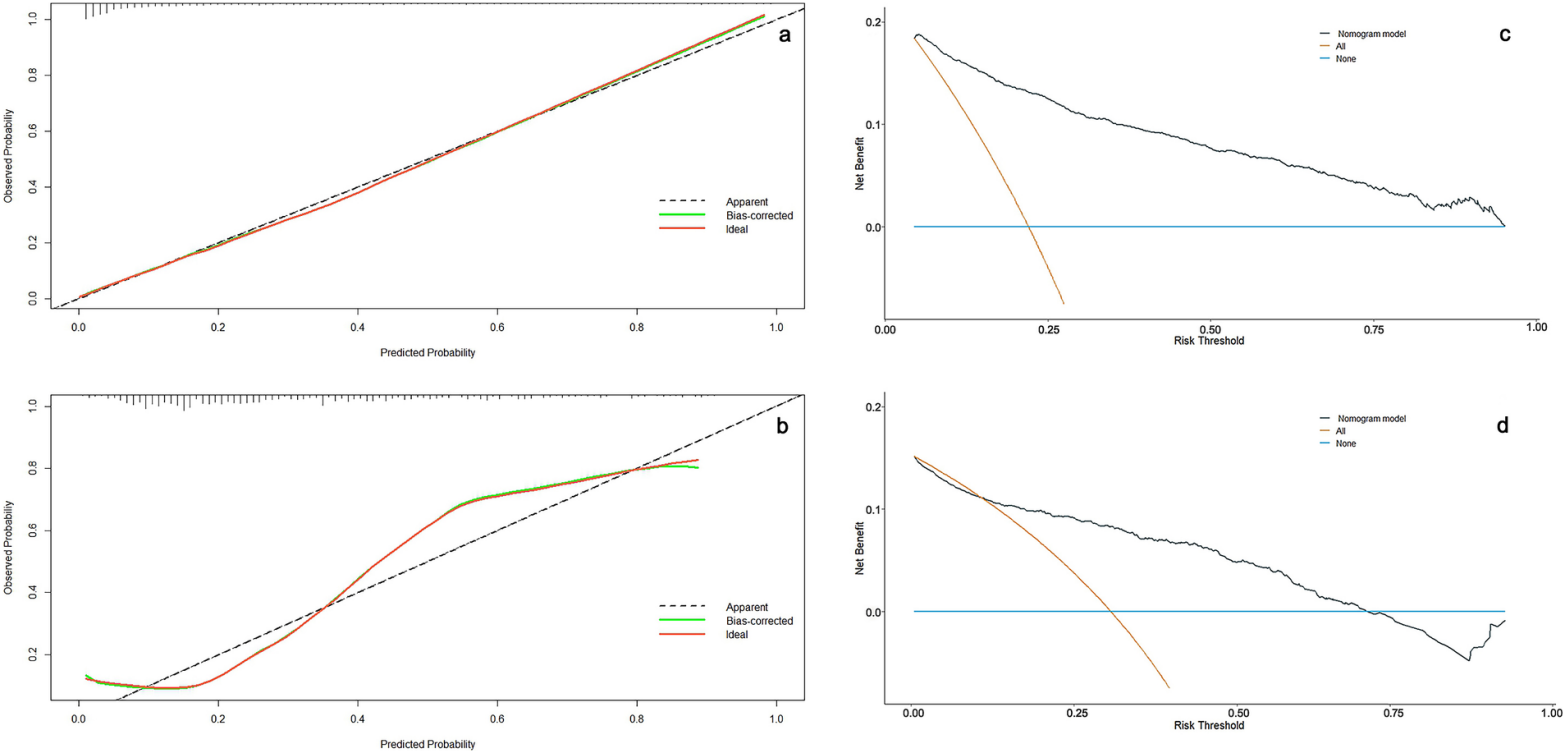
Nomogram calibration and decision curve for 31-day unplanned readmission of ischemic stroke. **(a)** Calibration curves for the nomogram model in the training set. **(b)** Calibration curves for the nomogram model in the validation set. The *x*-axis represents the predicted risk and the *y*-axis represents the actual observed. **(c)** Decision curves for the nomogram model in the training set. **(d)** Decision curves for the nomogram model in the validation set. The dark green line shows the risk model. The brown line indicates a 31-day unplanned readmission forecast for all patients. The blue line indicates a 31-day unplanned readmission forecast for no patients.

For the training set, the AUC was 0.883 (95% CI = 0.867–0.899), with a sensitivity of 81.31% and a specificity of 79.29%; for the validation set, the AUC was 0.817 (95% CI = 0.777–0.858), with a sensitivity a sensitivity of 77.40% and a specificity of 77.20%, as shown in Figures 2a,b. The calibration curves of the training set and the verification set tend to be close to the ideal curve, and the actual values agree well with the predicted values (Figures 3a,b). Additionally, the decision curve analysis revealed that the nomogram model yielded great net benefits in a risk threshold range of 0–1 in the training set, and a risk threshold range of 0–0.7 in testing set (Figures 3c,d).

## 4 Discussion

Readmissions are an internationally recognized measure of the quality of medical treatment and care (9–11). Hospital readmissions, especially unplanned readmissions, are considered to be outcomes that are not easily predicted (12). Because unplanned readmissions shortly after discharge are usually considered to be the result of unresolved issues at the time of discharge (5). As one of the most common clinical diseases, ischemic stroke not only has a high incidence, but also a serious condition, which imposes economic and health burdens on patients, society and health service systems around the world (13). Due to the high disease burden of ischemic stroke, it is important to study the relationship between stroke and hospital readmissions.

In recent years, the development of microsurgery has made great progress in the treatment of ischemic stroke, and the prognosis of patients has been greatly improved. However, short-term readmission rates after stroke remain high, with the 31-day readmission rate being around 15% in developed countries (14, 15). In this study, we investigated factors associated with 31-day readmission rate among patients with stroke in China. Among 3,035 patients with ischemic stroke, 22.04% had an unplanned readmission within 31 days, and it was comparable with Qiu’s et al. (16) report.

Complications not only increase the risk of ischemic stroke, but also affect the prognosis. Atrial fibrillation is the most common type of arrhythmia, and its association with ischemic stroke has been widely studied. Benjamin et al. (17) showed that patients with atrial fibrillation had a five-fold increased risk of ischemic stroke and a two-fold increased mortality. However, the relationship between atrial fibrillation and unplanned readmission in patients with ischemic stroke is rarely reported. Our findings showed that stroke patients with atrial fibrillation had a 1.887-fold increased risk of unplanned readmission, and indicated that clinicians should pay attention to the treatment and control of atrial fibrillation while treating stroke. China is a big tobacco country, and about one-third of the world’s tobacco is consumed by people in China, which is dominated by men (18). A number of studies have confirmed that smoking can cause a variety of diseases, mainly including cardiovascular disease, respiratory disease, cancer (19, 20). Smoking not only increases the risk of stroke, but also has a strong dose-dependent relationship (21). Chen et al. (22) reported that smoking could increase the risk of stroke recurrence in patients with cerebral infarction and transient ischemic attack, which was similar to our study results. Education of junior high school and below have an increased risk of unplanned readmission after stroke compared to education of high school and above. A prospective cohort study showed that low educational attainment was associated with an increased risk of stroke in both men and women (23). Moreover, a evidence from epidemiology and Mendelian randomization study reported that education had a protective effect on cardiovascular disease outcomes (24). Why educational attainment reduces stroke readmissions. Considering the reasons, it may have to do with the broad benefits of education, which higher levels of education are associated with healthier lifestyles/attitudes towards health care, safer working conditions and better health care. Medical staff should increase the frequency and time of health education in the face of patients with low education level. In order to improve the patients and their families to the disease of the degree of attention, and then improve the patients to the absence of cerebral infarction disease and recurrence knowledge. Patients with a stay of >16 days had a higher risk of unplanned readmission than patients with a stay of ≤16 days. A random forest algorithm research reported that the risk of readmission was low in patients (stroke) hospitalized for about 10 days, and longer or shorter length of hospital stay increased the risk of readmission (25). Studies in South Korea and the United States have reached similar conclusions (5, 15). The relationship between the length of hospital stay and readmission of stroke patients may be related to various factors such as the region, the type of disease studied, disease severity, and the level of medical treatment and nursing.

Among laboratory measures associated with ischemic stroke, our results showed that Hcy and UA were independent risk factors for unplanned reentry. A large number of previous studies have reported that Hcy is an independent risk factor for the occurrence and recurrence of ischemic stroke (26, 27). A meta-study found that high Hcy levels were positively associated with early hemorrhage transformation after IS, with no racial differences (28). UA has the dual properties of antioxidant and oxidation, on the one hand as an effective antioxidant can remove free radicals in the blood; On the other hand, oxidative stress can cause endothelial dysfunction and lead to cardiovascular diseases such as stroke (29). At present, the relationship between serum UA levels and stroke is also controversial. Sun et al. (30) found that UA therapy in patients with acute ischemic stroke can reduce the level of markers of oxidative stress. In contrast, a meta-study of 33,580 stroke patients showed (31) that increased UA levels increased the incidence and recurrence of IS, and there was no evidence to support a neuroprotective effect of UA. Han et al. study showed (32) that increased UA levels were associated with recurrence of IS and increased readmission risk of patients, which was similar to our study results.

Based on regression analysis, the nomogram prediction model integrates multiple independent risk factors together, and shows the proportion of the score by line segment on the same plane according to a certain probability (33, 34). At present, the nomogram model has been widely used in clinical medicine, especially in oncology (35). In this study, a nomogram prediction model was established based on the results of logistic multivariate regression analysis, and the area under ROC curve was 0.883 (95% CI = 0.867–0.899), indicating that the nomogram model had a good predicted values. The calibration curve showed that the nomogram model had good prediction accuracy. The decision curve results showed that the nomogram model had a higher clinical net benefit rate.

Limitations also exist in this study. Firstly, although this study is a large sample study, there may be some bias in the single-center, retrospective study. Secondly, although we studied the relationship between smoking and readmission in stroke patients. However, due to the retrospective study of this study, it is difficult for us to obtain the specific daily smoking amount of patients, so we cannot further study the relationship between smoking dose and readmission. Thirdly, medication adherence after discharge may also affect the incidence of recurrent ischemic stroke, our study did not analyze the effect of medication adherence. We will conduct a prospective, multicenter study to further validate the current model.

## 5 Conclusion

In this study, with 22.04% of ischemic stroke patients being readmitted within 31 days after discharge. Atrial fibrillation, smoking, education of junior high school and below, length of stay >16 days, Hcy, and UA were the most influential factors for readmission within 31 days. The nomogram prediction model based on the above risk factors has better predictive power, discernment, and higher clinical net benefit rate.

## Data Availability

The original contributions presented in the study are included in the article/supplementary material, further inquiries can be directed to the corresponding author.
